# A Multistage Heterogeneous Stacking Ensemble Model for Augmented Infant Cry Classification

**DOI:** 10.3389/fpubh.2022.819865

**Published:** 2022-03-24

**Authors:** Vinayak Ravi Joshi, Kathiravan Srinivasan, P. M. Durai Raj Vincent, Venkatesan Rajinikanth, Chuan-Yu Chang

**Affiliations:** ^1^School of Information Technology and Engineering, Vellore Institute of Technology, Vellore, India; ^2^School of Computer Science and Engineering, Vellore Institute of Technology, Vellore, India; ^3^Department of Electronics and Instrumentation Engineering, St. Joseph's College of Engineering, Chennai, India; ^4^Department of Computer Science and Information Engineering, National Yunlin University of Science and Technology, Yunlin, Taiwan; ^5^Service Systems Technology Center, Industrial Technology Research Institute, Hsinchu, Taiwan

**Keywords:** baby cry, feature vectors, MFCC, spectrograms, stack-based algorithms

## Abstract

Understanding the reason for an infant's cry is the most difficult thing for parents. There might be various reasons behind the baby's cry. It may be due to hunger, pain, sleep, or diaper-related problems. The key concept behind identifying the reason behind the infant's cry is mainly based on the varying patterns of the crying audio. The audio file comprises many features, which are highly important in classifying the results. It is important to convert the audio signals into the required spectrograms. In this article, we are trying to find efficient solutions to the problem of predicting the reason behind an infant's cry. In this article, we have used the Mel-frequency cepstral coefficients algorithm to generate the spectrograms and analyzed the varying feature vectors. We then came up with two approaches to obtain the experimental results. In the first approach, we used the Convolution Neural network (CNN) variants like VGG16 and YOLOv4 to classify the infant cry signals. In the second approach, a multistage heterogeneous stacking ensemble model was used for infant cry classification. Its major advantage was the inclusion of various advanced boosting algorithms at various levels. The proposed multistage heterogeneous stacking ensemble model had the edge over the other neural network models, especially in terms of overall performance and computing power. Finally, after many comparisons, the proposed model revealed the virtuoso performance and a mean classification accuracy of up to 93.7%.

## Introduction

Globally, around 130 million infants are born every year. Taking good care of babies is a major challenge, particularly in the initial stages of parenting. Many ways and ideas are available in many books and resources, but they do not provide impactful insights into resolving the issues. The principal reason is that it is hard to comprehend the meaning of infant's cries. Newborn children communicate with the world through crying. Experienced guardians, parental figures, specialists, and medical attendants comprehend the cries depending on their experience. Young parents get baffled and experience difficulty calming down their infants since all cry signals sound very similar to them. The major problem faced by many new parents is that they hardly understand the reason for the infant's cry (1–8). It is not possible to identify the reason just by looking at the face or analyzing the emotions of the infant (9–12). Many doctors believe that the reason for the infant's cry is based mainly on the patterns of their voice. If the parents do not know the main reason or the root cause of the infant's cry, then they would not be able to provide the required treatment. So, it would be highly beneficial if significant experiments were performed on the infants' audio signals. Their tone and pitch contribute a lot to finding better results. To find an efficient solution to this problem, a primary emphasis needs to be made on the voice patterns. The voice/audio signals generated by the babies contain many feature vectors that can be used in various deep learning or ensemble models. Machine learning systems possess automated learning capabilities, and their performance improves based on their previous experience devoid of any explicit programming (13–27).

Many convolutional neural network variants, like VGG16, VGG19, and AlexNet, mainly deal with the problems of classifying the results based on the features present in the audio signals. The extracted feature vectors contain much valuable information about the pitch, tone, and amplitude. These can help derive the entropy, energy, and spectral intensity of the audio signals.

Newborn child cry examination expands toward the auditory requirements of the infant's cry signals. Many mechanisms have been exploited for the infant cry order, lumber, and Mel-recurrence cepstral coefficient (MFCC). Using a wide variety of elements to perceive and arrange newborn child crying remains somewhat troublesome, even as vulnerability exists regarding which of these elements is pertinent.

The proposed model makes use of the MFCC algorithm in the initial stages. In strong handling, the Mel-recurrence cepstrum portrays the transient power range of a sound based on a linear cosine change of a log power range on a non-linear Mel scale of recurrence. MFCCs are coefficients that collectivelymake up an MFCC. This is mainly required in the data preprocessing phase because the dataset comprises the audio signals. To perform mathematical calculations and to compute the results, it is vital to convert the audio signals into a 2D feature vector so that various deep learning and advanced classification models can be applied to predict the results.

Our proposed work reveals the following major contributions:

- We performed data preprocessing and generated the spectrograms of the audio signals.
- We worked on the major CNN variants like VGG16 and Yolov4 and used transfer learning approaches to perform multiclass classification. While using the pretrained weights of the models, excessive hyperparameter tuning was performed to enhance the performance of the models.
- A distinctive comparison was made between the CNN variants in terms of accuracy, computational time, and resources. This resulted in Yolov4 being the best model among others. It achieved an accuracy of 75%.
- To improve the performance of the model and provide a more efficient solution, a multistage heterogeneous stacking ensemble model was devised. This model made use of the major ensemble-based boosting algorithms to increase the accuracy and other evaluation metrics of the model.
- On analyzing the cognitive performance of major classifiers, Nu-support vector classification (NuSVC), Random Forest (RF), XGBoost, and AdaBoost were selected for this task. NuSVC is like SVC but uses a parameter to control the number of support vectors. It is based on the implementation of LIBSVM. RF is an outfit learning technique for grouping and relapsing different assignments that work by developing numerous choice trees at the preparing time. For order assignments, the result of RF is the class chosen by most trees. XGBoost is an execution of slope-supported choice trees intended for speed and execution. The AdaBoost algorithm, short for adaptive boosting, is a boosting procedure used as an ensemble method in machine learning. It is called adaptive boosting because the loads are reallotted to each occurrence, with higher loads doled out to inaccurately ordered examples. The proposed multistage heterogeneous stacking ensemble model achieved an accuracy of 93.7%.

## Related Work

During the 2000s, most techniques employed in newborn child research were identified with neural organizations, including the scaled form (5). Their review included the details about applying many neural network models and traditional machine learning algorithms like KNN and SVM to predict the reason for the infant's cry. Considering a unique circumstance, the work in (28) zeroed in on making a programmed framework that could recognize diverse newborn child needs dependent on crying. It separated different arrangements of paralinguistic highlights from the child cry sound signals and prepared different rule-based or measurable classifiers.

The work in (29) developed an NonLinear Forcasting (NLF) model that includes the Euclidean distance for its goal work, which is normally a unique instance of difference. In addition, it often experiences slow intermingling. This review proposes a summed up and quick uniting non-negative dormant variable [a generalized and fast-converging non-negative latent factor (GFNLF)] model to resolve these issues. Its primary thought is two-fold: (a) taking on—dissimilarity for its goal work, subsequently improving its portrayal capacity for Host Based Intrusion Detection System (HiDS) information; (b) concluding its energy joined non-negative multiplicative update calculation, along these lines accomplishing its quick intermingling. Experimental investigations on two HiDS grids rising out of genuine RSs show that, with cautiously tuned hyperparameters, the GFNLF model outperforms groundbreaking models in both computational effectiveness and expectation exactness for missing information in a HiDS lattice.

The research in (4) developed a time–frequency-based analysis called STFT. A total of 256 discrete Fourier transform focuses were considered to figure out the Fourier change. It accomplished a deep convolutional neural organization called AlexNet with a few improvements to group the recorded newborn child cry. To work on the viability of the previously mentioned neural organization, stochastic gradient descent with momentum (SGDM) was used to perform the calculation.

The authors in (6) obtained and broke down sound elements of infant's cry signals in schedule and recurrence areas. In view of the connected elements, we can arrange cry signals to clear cry implications for cry language acknowledgment. Highlights separated from sound component space incorporate linear predictive coding, linear prediction cepstral coefficients, Bark frequency cepstral coefficients, and MFCCs. Packed detecting method was used for characterization, and useful information was used to plan further and confirm the proposed approaches. Tests showed that the proposed infant's cry detecting approaches offer accurate and promising outcomes.

The work in (7) portraying the advancement of significant information innovation, anticipating clients' buying goals through precise information of their buying practices has turned into a fundamental system for organizations to perform accuracy promotion and increase deal volume. The information of clients' buying behavior is described by an enormous sum, significant changeability, and long haul reliance. Along these lines, the bidirectional long short-term memory (BiLSTM) model is used in this article to examine the client's buying behavior. First, the model accepts client ID as the benchmark of grouping, catching the variance law of the client purchase volume and completely mining the drawn-out reliance of client's buying behavior. Second, the BiLSTM model adaptively extricates highlights, figures out the “start to finish” forecast of client's buy behavior, and diminishes the design subjectivity. This article checks the viability of this strategy depending on the genuine client buying behavior informational indexes. The investigation results show that the BiLSTM technique has high precision in examining the client's buying behavior.

The significant goal of this exploration work (18) was to introduce another procedure to recognize cancer. The proposed engineering precisely divided and characterized harmless and dangerous cancer cases. Diverse spatial area techniques to improve and precisely divide the information pictures were applied. Also, AlexNET and GoogLeNet were used for characterization, wherein two score vectors were acquired. Further, both score vectors were melded and provided to many classifiers alongside the Softmax layer. Assessment of this model is done on top medical image computing and computer-assisted intervention (MICCAI) challenge datasets, i.e., multimodal brain tumor image segmentation 2013, 2014, 2015, and 2016 and ischemic stroke lesion segmentation 2018 separately.

The work in (30) emphasized the complete exploration plans to arrange baby's cries into their social characteristics by utilizing evenhanded and insightful AI approaches. Toward this objective, the authors have considered customary AI and later profound learning-based models for child cry arrangement using acoustic elements, spectrograms, and a mix of the two. They have performed a point-by-point experimental review of the open access corpus and the CRIED dataset to feature the adequacy of fitting acoustic elements, signal processing, or AI procedures for this purpose. Major work was done by presuming that acoustic elements and spectrograms together will bring better outcomes. As a side outcome, this work additionally underscored the test of a deficient child cry data set in displaying baby's behavioral attributes.

This study (31) investigates a neural transfer learning way to create precise models for recognizing babies that have experienced perinatal asphyxia. Specifically, the authors have investigated the speculation that portrayals obtained from grown-up discourse could educate and further develop execution based on models created on newborn baby discourse. Their tests show that models depending on such portrayal moves are resilient to various kinds and levels of commotion, just as to flag misfortune on schedule and recurrence areas. The work analyzes the exhibition of a residual neural organization. Their ResNet model was pretrained on a few discourse assignments in characterizing perinatal asphyxia. Among the implemented models, the model for the word recognition task performed the best, recommending that the varieties learned for this undertaking are generally closely resembling and helpful to their objective assignment. The support vector machine prepared straightforwardly on MFCC highlights ended up being a solid benchmark and, assuming fluctuation in forecasts was of concern, a favored model.

In this article (32), the authors present a safe medical care framework that performs an acoustic examination of messy boisterous baby cry signs to concentrate and gauge specific cry attributes quantitatively and group strong and weak babies indicated only by their cries. In the lead of this infant cry-based indicative framework, the unique MFCC highlights as well as static MFCCs are chosen and removed for both expiratory and inspiratory cry vocalizations to deliver a discriminative and instructive component vector. Then, the authors made a remarkable cry design for each cry vocalization type and neurotic condition by presenting a clever thought utilizing the boosting mixture learning (BML) technique to infer either sound or pathology subclass models independently from the Gaussian mixture model-universal background model. Also, a score-level combination of the proposed expiratory and inspiratory cry-based subsystems was performed to settle on a more dependable choice. The trial results show that the adjusted BML strategy has lower error rates than the Bayesian methodology when considered as a kind of perspective technique.

## Data Preparation

Data processing is the first step in designing the model. The dataset comprises several cry signals that have been taken from the National Taiwan University Hospital—Yunlin Branch for research. These signals correspond to the reason for the infants' cry. These four reasons are hunger, pain, tiredness, and diaper. The major steps involved in the preprocessing phase are data cleaning, audio scaling/normalizing, framing, windowing, and later spectrogram formation. In this article, the baby cries were acquired from the Division of Obstetrics and Gynecology at the National Taiwan University Hospital Yunlin Branch, Taiwan. They encountered no inconveniences during birth, and their introduction to the world loads, ages, and gestational ages was without neurotic discoveries. Their age was somewhere between 1 and 10 days. Also, all of the baby cries were recorded using a SONY HDR-PJ10 HD computerized video recorder with an underlying mouthpiece, Sony Corporation, Tokyo, Japan. The cries of newborn children were recorded in a supine position. The amplifier was held around 40 cm from the newborn child's mouth. Each recording document lasts somewhere between 10 and 60 s. Figure 4 illustrates the workflow of the proposed models.

### Preprocessing

**Figure 1:**
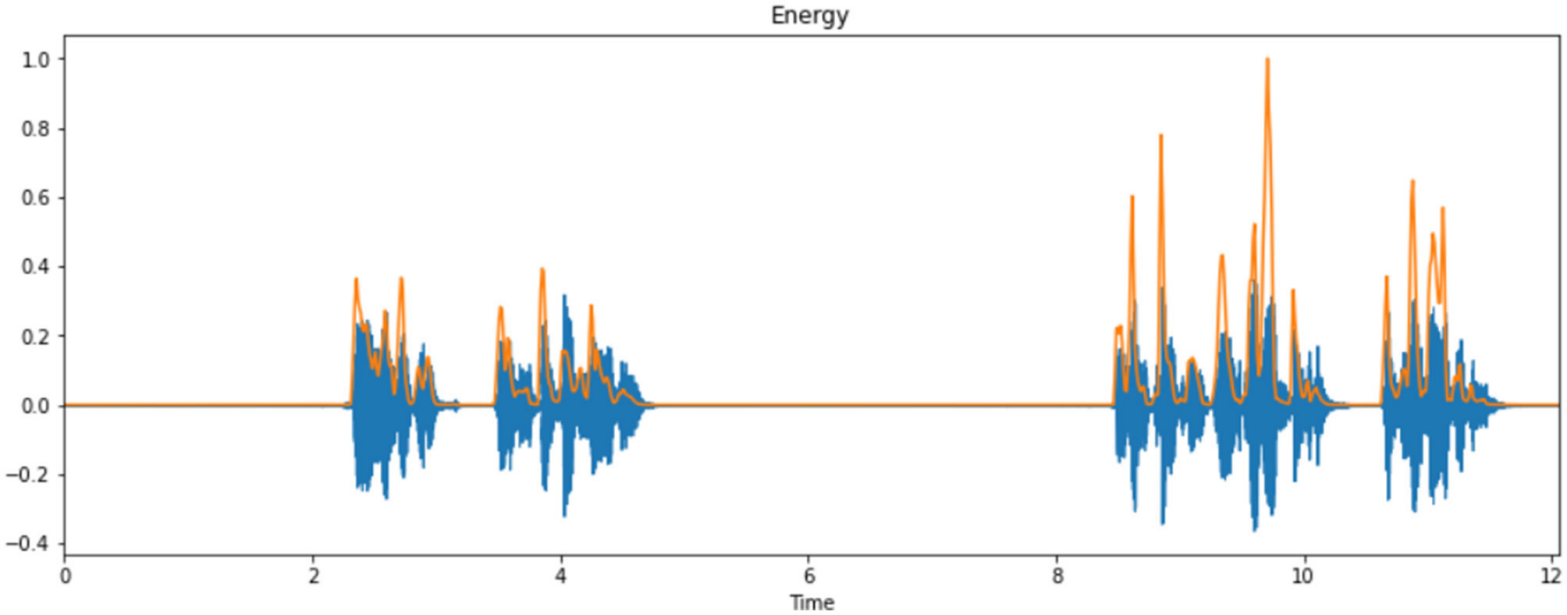
Infant cry acquisition setup and analysis.

**Figure 2:**
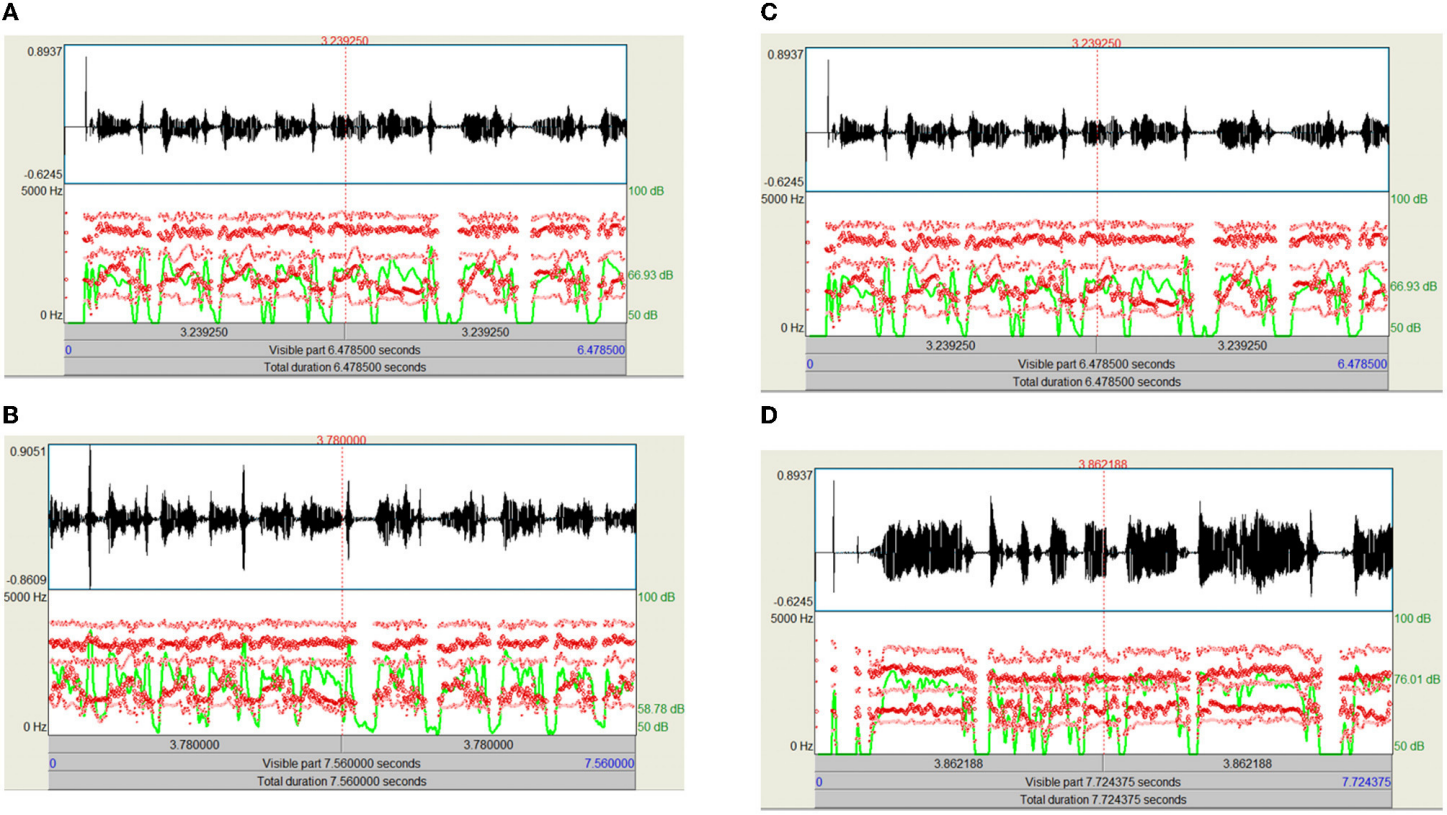
**(A)** Audio signal of a sample baby cry (diaper) with intensity (green) and formant information (red), **(B)** audio signal of a sample baby cry (hunger) with intensity (green) and formant information (red), **(C)** audio signal of a sample baby cry (pain) with intensity (green) and formant information (red), and **(D)** audio signal of a sample baby cry (sleep) with intensity (green) and formant information (red).

**Figure 3:**
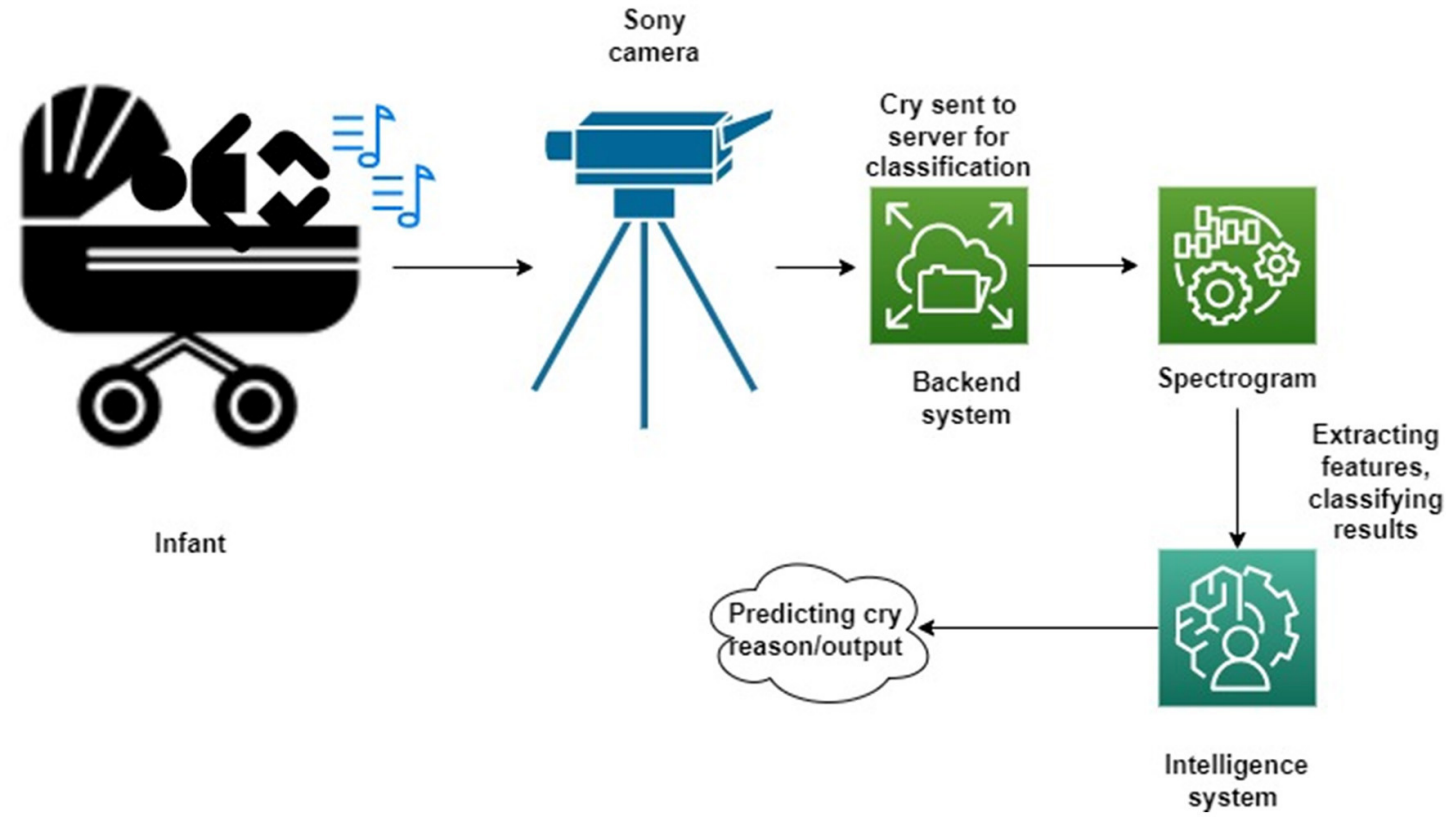
Audio signal of a sample baby cry.

In this phase, all of the input infant cry audio files are preprocessed. It basically involves applying a double loop on the entire dataset. Here, the noises and other disturbances from the audio files are also removed to get better results. This is because if we train our model on the dataset, which contains noise or unwanted data, then the model might have some significant characteristics and might make wrong interpretations. The algorithm basically converts the input audio files to various keyframes, which are then converted into a series of feature vectors. So, every audio keyframe would have a feature vector, which when combined would cause a 2D feature vector containing the numerical values of all of the relevant features of the audio signal. MFCC is utilized to separate the interesting components of discourse tests. It addresses the momentary force range of human discourse. The MFCC method uses two sorts of channels:directly dispersed channels and logarithmically separated channels. To catch the phonetically significant attributes of discourse, the sign is communicated on the Mel-recurrence scale. The Mel scale is mostly established on examining the pitch or repeat saw by the human. Subsequently, the scale is wrapped up into the units of Mel. The Mel scale is commonly an immediate preparation under 1,000 Hz and logarithmically isolated over 1,000 Hz. MFCC involves six computational advancements (3). This progression likewise includes passing the sign through the channel, which highlights higher recurrence in the frequency band. It likewise underlines the extent of some higher frequencies in regard to other lower frequencies to improve the energy. Librosa is a library available in Python that reads and processes the audio signals. Figure 1 represents the sample audio signal of an infant's cry. Figures 2A–D illustrate the audio signal of a sample baby cry for diaper, hunger, pain, and sleep, respectively. Figure 3 illustrates the infant cry acquisition setup and analysis.

**Figure 4:**
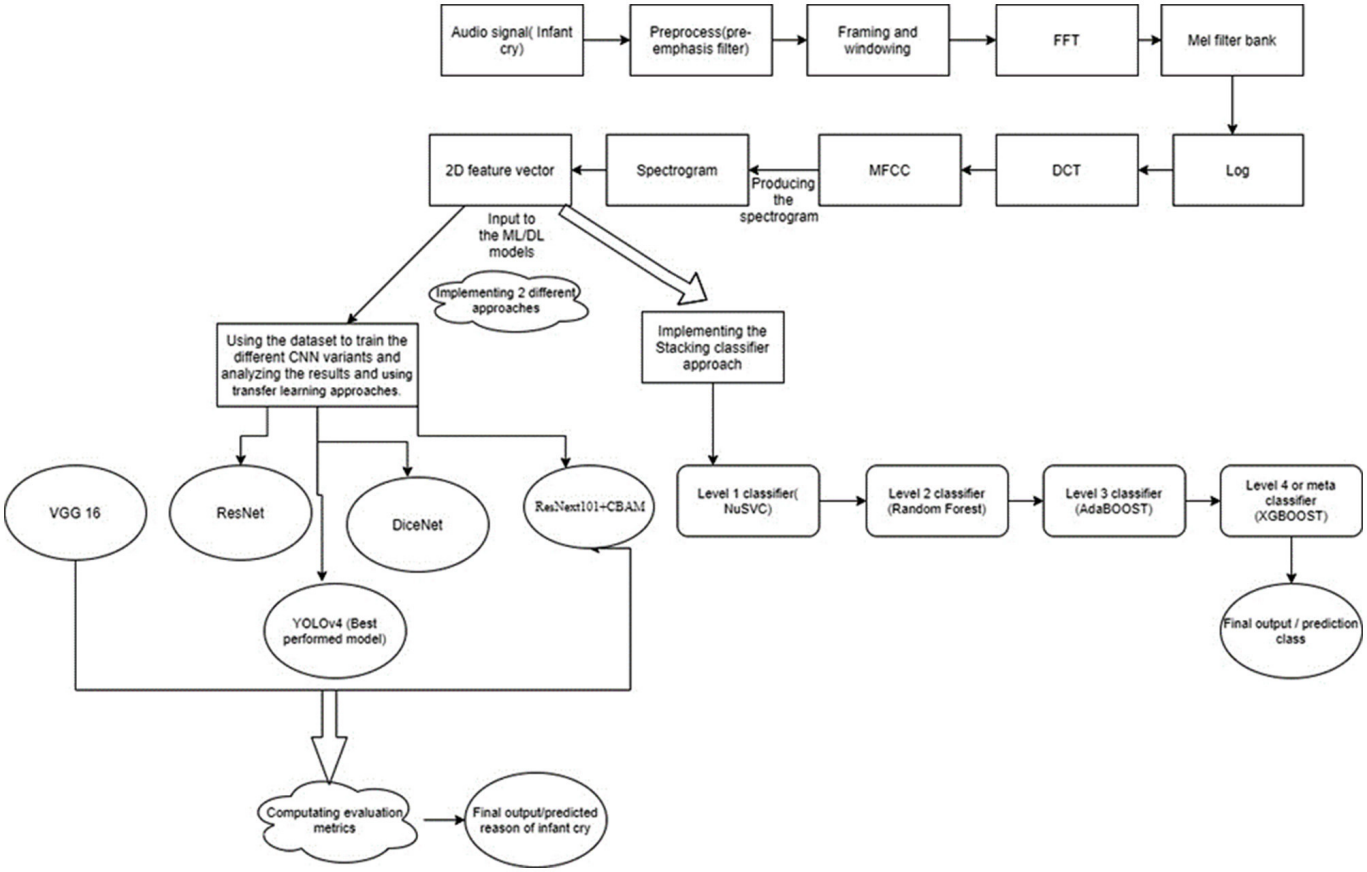
Workflow of the research techniques used in this work.

### Frequency Feature Extraction

It pays attention to the recurrence parts of the sound sign. Signals are changed from the time–space to the frequency area using the Fourier transform. Band energy proportion, spectral centroid, and otherworldly motion are models.

### Spectral Roll-Off

Roll-off is the steepness of a transfer function with frequency, particularly in electrical network assessment, and most especially with regard to direct circuits in the advancement between a passband and a stopband. It is mostly applied to the expansion loss of the network but can, on a fundamental level, be applied to any appropriate function of frequency, and any technology, not just devices. It is customary to check roll-off as a part of logarithmic frequency; consequently, the units of roll-off are either decibels per decade (dB/decade), where a decade is a 10 times increase in frequency, or decibels per octave (dB/8ve), where an octave is a 2-fold increase in frequency.

**Figure 5:**
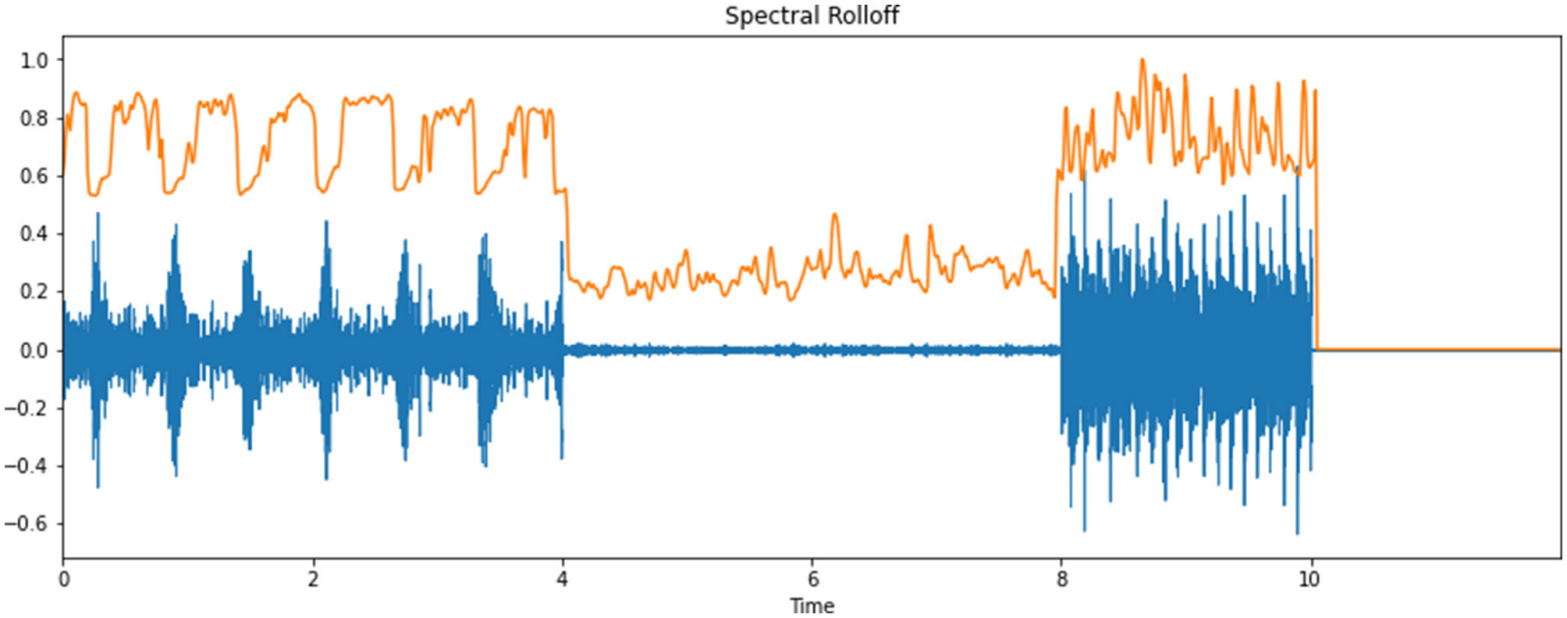
Spectral roll-off of an infant cry's audio signal.

Roll-off comes from the fact that in many networks roll-offs tend toward a constant gradient at frequencies well-away from the cut-off point of the frequency curve. Roll-off enables the cut-off performance of such a channel network to be diminished to a single number. Note that roll-off can occur with diminishing frequency as well as as increasing frequency, depending on the bandform of the channel being considered: for instance, a low-pass channel will roll-off with increasing frequency, but a high-pass channel or the lower stopband of a band-pass channel will roll-off with reducing frequency. For conciseness, this article depicts only low-pass channels. This is to be taken in the spirit of model channels; comparative guidelines may be applied to high-pass channels by trading articulations, for instance, “above cut-off frequency” and “below cut-off frequency. Figure 5 illustrates the spectral roll-off of an infant cry's audio signal.

### Spectral Centroid

**Figure 6:**
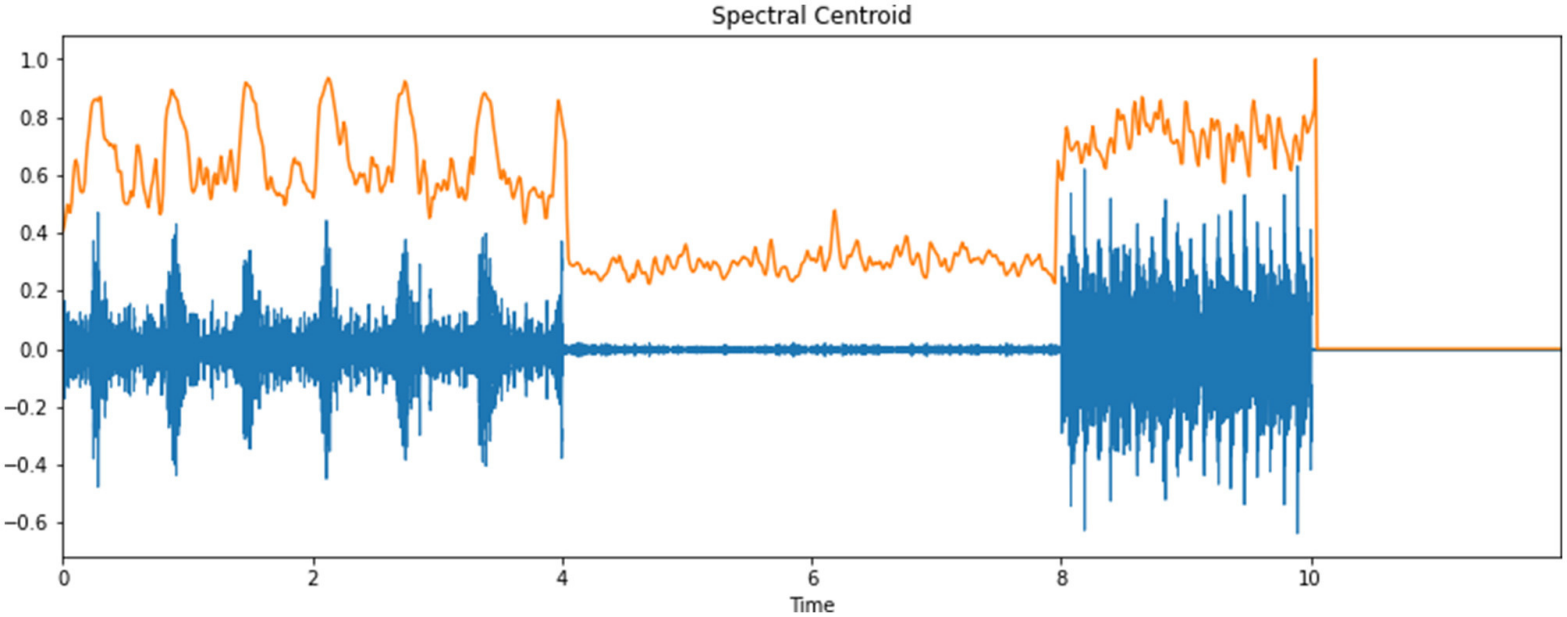
Spectral centroid of an infant cry's audio signal.

The spectral centroid is a measure that shows the location of the “focal point of mass” of the spectrum. Perceptually, it has a strong association with the impression of “splendor” of a sound and hence is utilized to portray melodic tone. Figure 6 portrays the spectral centroid of an infant cry's audio signal.

### Spectral Flux

**Figure 7:**
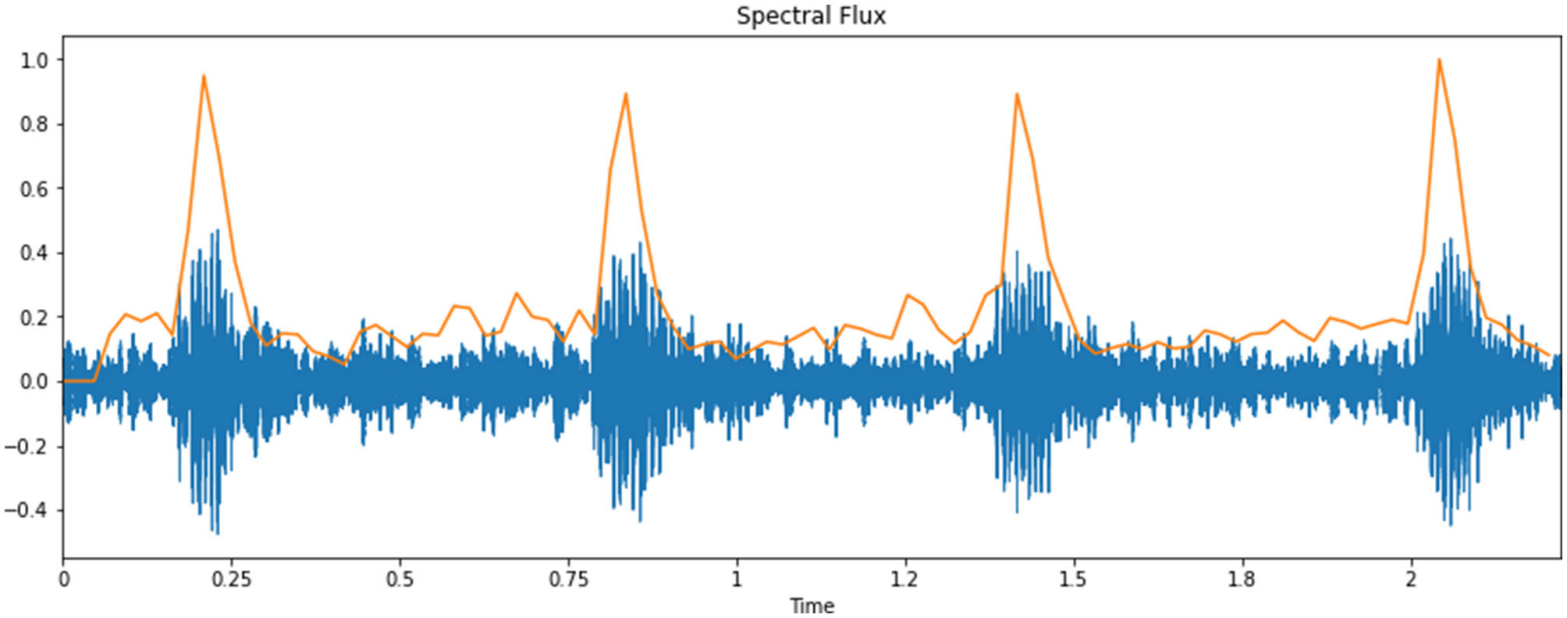
Spectral flux of an infant cry's audio signal.

The spectral flux/transition is a helpful measure for differentiating signals whose spectrum changes slowly from signals whose spectrum changes rapidly. It has a lower incentive for the previous “slow” class of signals and a higher incentive for the later “quick” class of signals. Figure 7 shows the spectral flux of an infant cry's audio signal.

These non-linear features help us analyze the patterns and frequency domains of the audio signals. The values derived from these feature vectors would help decide the appropriate methodologies suitable for the data preprocessing phase.

### Framing and Windowing

Framing is the process of segmenting the entire audio signal into small frames of a fixed size. A single audio file is converted into *N* number of frames so that the algorithms work better. Because a single audio file would contain many feature vectors, this would become highly complicated to derive the results. The adjacent frames are also separated by a specific gap so that they do not overlap. Setting a space of 256 or 512 is appropriate for the MFCC process to avoid overlaps.

### Fast Fourier Transform

It performs the task of conversion of each single frame of the audio keyframes from the phase of the time domain to the frequency domain, as most of the calculations are performed in this domain.

### Mel Filter Bank

There are many frequencies ranges in fast Fourier transform. The most significant one is enormously inclusive, and voice signals do not trail the unswerving gauge.

### Discrete Cosine Transform (DCT)

**Figure 8:**
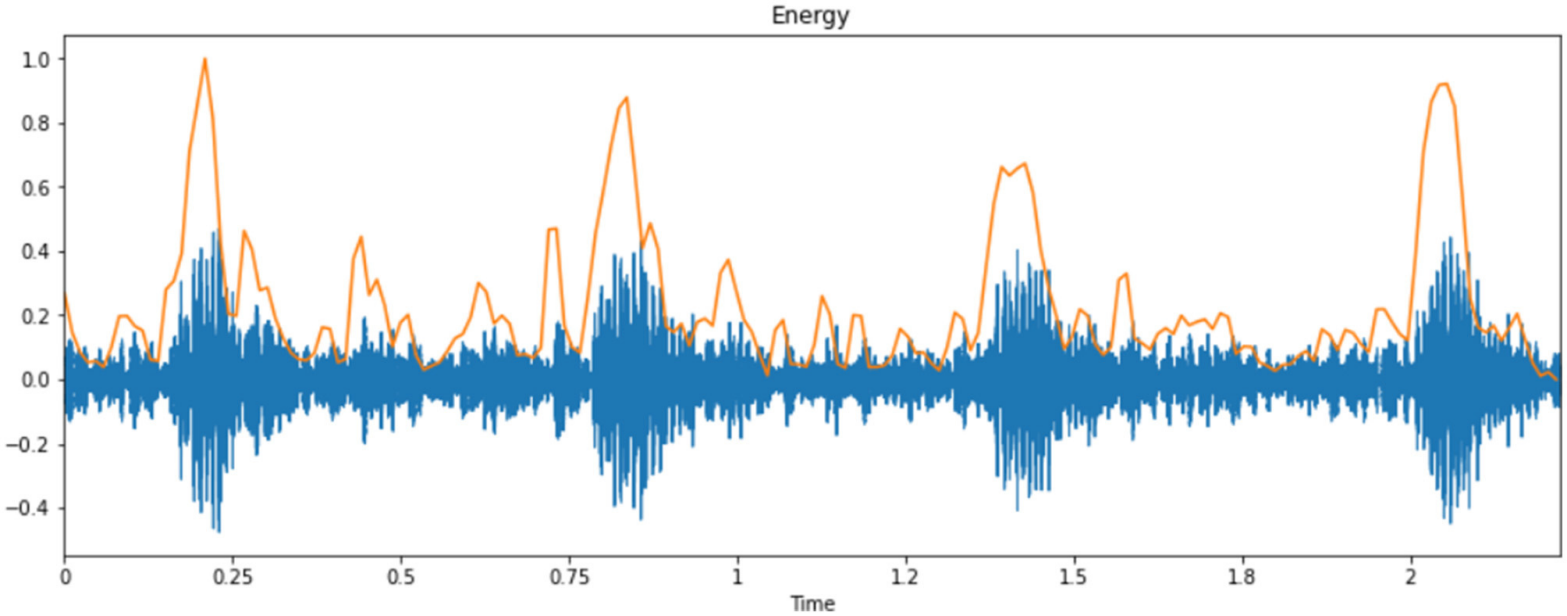
Energy of a sample cry spectrogram.

This process involves a cycle that can transform the log Mel spectrum into time domain. The outcome generated by the process is defined as MFCC. The set of coefficients is called acoustic vectors. According to this concept, all of the information values are transformed into a segment of auditory vectors. Figure 8 represents the energy of the cry spectral waveform.

## Applying CNN Variants On The Cry Spectrograms

After data preprocessing and generation of the spectrograms and tabular 2D dataset, we applied the various CNN variants. The expected workflow comprises the following steps.

### Data Distribution

First, the dataset was split into training and test sets in the ratio of 80:20. In total, 80% of the entire dataset that comprises the feature vectors of the spectrograms was used for training the different models. The training, testing, and validation accuracies of both the proposed models were compared and analyzed.

Table 1 consists of the number of testing samples for each prediction class. The data distribution sampling is the same for YOLOv4 and VGG16.

**Table 1:** Data distribution for different classes.

**Class**	**Number of training samples**	**Number of testing samples**
Sleep	12,820	3,205
Hunger	12,980	3,245
Pain	14,740	3,685
Diaper	14,204	3,551

### Implementing VGG16

The main reason for using VGG16 was its merits in predicting the multiclass outputs of the model (6). According to various research studies, it is one of the best CNN variants that utilizes the transfer learning approaches for multiclass classification problems.

Table 2 represents the information about the layers and the size of the kernels. Table 3 presents the summary of the VGG16 model, and Table 4 lists the hyperparameter settings of the VGG16 model.

**Table 2:** Layer architecture of VGG16.

**Layers**	**Size**
A doubly linked convolution layer	It consists of 64 channels—each consisting of a kernel of size 3 × 3
A Maxpool layer	It consists of a kernel having a pool of size 2 ×2 and a stride of size 2 × 2
A doubly linked convolution layer	It consists of 128 channels, each of size 2 ×2
A Maxpool layer	It consists of a kernel having a pool of size 2 ×2 and a stride of size 2 × 2
A triply linked convolution layer	It consists of 512 channels, and each channel has a kernel of size of 3 × 3
A Maxpool layer	It consists of a kernel having a pool of size 2 × 2 and a stride of size 2 × 2
A triply linked convolution layer	It consists of 512 channels, and each channel has a kernel of size of 3 × 3
A Maxpool layer	It consists of a kernel having a pool of size 2 × 2 and a stride of size 2 × 2

**Table 3:** Parameters—VGG16.

**Layer type**	**Output size**	**Parameter**
Input layer	1 × 256 × 40	0
Convulated 2D layer	128 × 256 × 40	1,280
Batch normalization layer	128 × 256 × 40	512
Activation layer	128 × 256 × 40	0
2D Maxpool layer	128 × 256 × 8	0
Dropout layer	128 × 256 × 8)	0
bidirectional_1	Bidirection (None, 256, 32)	55,488
permute_1 (Permute)	(None, 256, 128, 2)	0
bidirectional_2 (Bidirection)	(None, 256, 32)	12,480
time_distributed_1 (TimeDist)	(None, 256, 32)	1,056
conv2d_3 (Conv2D)	(None, 128, 256, 4)	147,584

**Table 4:** Hyperparameters settings—VGG16.

**Name**	**Settings**
Nodes used per trained layers	1,024
Epochs	300
Optimizers	Adam and SGD
Lr—learning rate	0.0001
Lrd—learning rate decay	Yes
Drop out	0.25

After performing a lot of comparisons among the various hyperparameter selected values, the proposed VGG16 model was performing at its best on the above-mentioned ones. The observations made on these selected values can provide deep insight into the performance of the model. From this, we can infer that the nodes per trained layers and drop-out values contributed in the most significant way to the training of the entire model. The model was trained on 300 epochs. This was the tuned parameter; the epochs above 300 showed a degrading performance.

### Implementing YOLOv4

The accuracy of CNN should be determined through many provisions. The pragmatic testing of such element blends on enormous datasets is needed, similar to the hypothetical pursuit of the outcomes. A few viewpoints, for example, cluster standardizations, are simply enough to explicit inadequately spaced datasets; however, many others are rational to a larger part of representations, assignments, and datasets. To accomplish outstanding results, this proposed work utilizes original provisions, for example, Mish actuation and DropBlock regularization are some of them joining some of them.

### Hyperparameter Selection for YOLOv4

The Yolov4 model has 53 convolution layers of size 1 × 1 and 3 × 3 kernels. It also has 30 hyperparameters for improving the training and the performance of the model. Each hyperparameter has a specific potential to create a huge impact on the overall training and learning of the model. The following hyperparameters have been selected after understanding the architecture and learning the observations from various research articles using YOLOv4 (16). After many tests and evaluations, the final YOLOv4 model was subjected to the following hyperparameters. Table 5 represents the hyperparameter settings of the YOLOv4 model.

**Table 5:** Hyperparameter settings—YOLOv4.

**Name**	**Settings**
Batch size	10
Epochs	380
Optimizers	Adam
Lr—learning rate	0.001
Lrs—learning rate schedule	Learning rate increases by 0.1
Drop out	0.20
Early stopping	There is a decline in the validation loss for 50 epochs
Momentum	0.924
Weight decay	0.0005
Anchor_t (anchor-multiple threshold)	4.0
F1_gamma	0.2

### Implementing FastGCN

FastGCN is a graph-based model with a purposeful diagram model for semi-supervised learning approaches. Moreover, on account of the recursive neighborhood development of transverse layers, preparing huge and thick diagrams is time- and memory-consuming. This is a hybrid model, and its hyper tuning methodologies are the fundamentals of implanting capacities. Accordingly, it utilizes Monte Carlo strategies to dependably surmise the integrals, which prompts the group preparation plan.

### Comparing the Performance of the Top CNN Variants

The performances of top CNN models are compared in Table 6.

**Table 6:** Comparing the performance of top CNN models.

**Variant name**	**Year (developed)**	**Category**	**Role**	**Parameters**	**F1 score**	**Error rate**
VGG16	2014	Spatial exploitation and multiclass classification	Uses small-sized kernels and performs better for multiclass problems	140 M	~72.1%	ImageNet: 27.3%
ResNeXt10 + CBAM	2018	Consideration	Exploits both feature map and spatial information	48.76 M	73.4%	26.6%
ResNet	2016	Depth in learning using multi-path technique	Provides a mapping-based skip connection	25.63 M	72.8%	27.2%
YOLOv4	2020	Spatial exploitation	Performs multiclass classification using pretrained weights—one of the major transfer learning models	60 M	75.2%	24.8%
DiceNET	2021	Dimensional based	It performs dimensional-based CNN, including height, weight, and depth	20 M	74.5%	25.5%

The performance of these CNN variants was not up to the mark, as these could not handle some outliers even after performing hypertuning. All of these models had low computational power and did not yield high accuracy. It was required to go with another approach that would yield better accuracy and have fast computational power. These are some approaches that combine many ensemble-based algorithms in a stacked form. These algorithms have a higher chance of training the model at a lesser computational time and provide better performance. Table 7 presents the merits and demerits of the top CNN variants.

**Table 7:** Comparing the merits and demerits of the top CNN variants.

**CNN variant name**	**Merits**	**Demerits**
VGG16	Provides the benefits of effective approachable fields in training It also introduces the concept of a simple and homogeneous topology between various audio key frames	Its computational power is dependent on the fully linked layers and is very expensive
ResNeXt101 + CBAM	Provides flexibility in depth and dimensions It has a consistency of maintaining a large amount of data flow between hidden layers, which contribute to information gaining attributes	As the number of feature vectors is very high in this model, the number of parameters and their values will also need to be increased, leading to a slow learning rate
YOLOv4	It is twice as fast in computing results than other top CNN variants In terms of accuracy and other metrics, it provides better performance	Requires a well-scaled and normalized dataset, as its hypertuning variables have restrictive abilities in some cases
ResNet	Error rate reduces for complex architectures The gradient problems are resolved (6)	It has a complex architecture. It has overfitting of hyperparameters
DiceNet	These convolutions use highly enhanced feature filters in every layer The dimension-wise vectors are mixed proficiently It provides high-accuracy multiclass classification (13)	It may take a lot of computational time in prediction

## Multistage Heterogeneous Stacking Ensemble Classification Model For Higher Predictive Performance

The stacking approach is an ensemble learning technique that uses various classifiers at different levels to produce better outputs. The data are trained at different levels by different classifiers. Since our dataset contains spectrograms of different audio frames, it is convenient to use different classifiers as any one specific classifier will not give the correct output. This was the main reason the top CNN variants were not performing up to the mark and taking a lot of time in training (6).

The following proposed model consists of four different levels. Each level corresponds to one classifier, and the output produced by each level will contribute to the performance of the next levels.

### Motivation to Use the Stacking Approach

The major benefit of using the stacking approach is that it can use the features and benefits of the different boosting algorithms to a better extent. There are many boosting algorithms like XGBOOST and AdaBoost that perform much better compared to neural networks and other deep learning models. This is because top CNN variants were performing slowly due to the large dataset. Their process involves various recursive backtracking methods and solves overlapping subproblems. The highly advanced boosting algorithms avoid these recursive calls to some extent.

### Implementation

The major objective behind the working of the proposed model is based on the characteristics of the top four classifiers. It is important to choose such classifiers that would increase the accuracy of the model by consuming less computational resources, time, and power. The entire model consists of four levels. Each level corresponds to a classifier. Initially, the spectrogram is fed as input to the first level classifier. Each level has a share of 25%. The classification results of the first classifier are stored in a cache and taken forward to the next level classifier. The next level classifier learns and gets trained on the basis of the prediction outputs of the previous level classifiers. Due to this, they will always try to improve the accuracy of the model as compared to the previous stages. The third and fourth classifiers play a major role in determining the performance and accuracy of the model.

### Model 1

This model uses SVC, multilayer perceptron (MLP), NuSVC, and RF classifiers. These classifiers are accessible in Scikit-learn. The most reasonable tuning will be applied to this model. To put it obtusely, if some classifiers are underperforming, a pile of them would most likely be garbage as well. To produce appropriate results, the hyperparameters of each classifier are fixed. With this model, an accuracy of 82–83% was achieved.

### Model 2 (Improvement of Model 1)

This model will support ensemble learning algorithms like XGBOOST, AdaBOOST, RF, and NuSVC at four different levels. The performance and the accuracy produced by this model were comparatively better than the previous approach. The main reason for choosing the boosting algorithms over the traditional machine learning algorithms was that these algorithms are highly flexible and support the parallel processing technique. Parallel processing is very important when we have a large dataset because it avoids computing the results in a recursive manner. Unlike the deep learning models, they are faster than gradient boosting. They can perform cross-validation after every iteration (5). XGBoost utilizes choice trees as base students, joining numerous powerless students to make a solid student. Accordingly, it is alluded to as an outfit learning technique since it utilizes the yield of many models in the last forecast. XGBoost or extreme gradient boosting may be very well-placed into different use cases like positioning, ordering, relapsing, and client-characterized forecast issues. It is an ideal mix of programming and equipment advancement methods to yield common results by utilizing fewer processing assets in the briefest measure of time. With this approach, an accuracy of ~87% was achieved with 300 epochs.

### Data Distribution

The dataset is split into training and testing sets in the ratio of 75:25. This the distribution of the testing samples of all the classes. Table 8 represents the number of testing samples for each class.

**Table 8:** Number of testing samples for each class.

**Class**	**Number of training samples**	**Number of testing samples**
Sleep	12,924	4,308
Hunger	14,517	4,839
Pain	14,115	4,705
Diaper	14,418	4,806

### Hyperparameter Tuning in XGBOOST and AdaBoost

XGBoost is a highly advanced implementation of the gradient boosting algorithm. It provides a lot of flexibility to enhance the performance of the model. The main advantage offered by XGBoost is that it helps improve the training of the model. Also, the proposed deep learning models had some overfitting instances. These could not be removed or reduced due to the limitations in the parameters of the various hidden layers. The training speed can be increased by increasing the max_Depth value to some extent. We had to implement some trail-error methods to fix the best value of max_Depth. This variable has a huge impact on the training speed of the model. According to a research article (8), a learning rate of 0.0001 was considered to be the most optimal for the initial stage. Later, the learning rate was increased to 0.001 on making some relevant observations in training accuracy. The parameter named objective was set to multi:softmax. The main reason for considering this value was the structure of our dataset. The dataset had multiclass output; hence, using multi:softmax was considered appropriate. Since XGBoost can predict the output much better than AdaBoost in terms of accuracy and precision, it is used in the last or fourth level of the proposed architecture.

The AdaBoost algorithm has a better learning rate and better performance among multiple *k*-fold cross validations. When fitting the last model, it could be attractive to either expand the number of trees until the difference of the model is decreased across rehashed assessments or to fit numerous last models and normalize their expectations. A significant hyperparameter for AdaBoost is *n*_estimator. By changing the number of base models or frail students regularly, we can change the precision of the model. The number of trees added to the model should be high for the model to function admirably, frequently hundreds, if not thousands. After all, the more the number of feeble students, the more the model will change from being high one-sided to low one-sided. The learning rate relies profoundly on the number of *n*_estimators. Naturally, it is set to 1, yet it tends to be expanded or diminished depending upon the assessors utilized. For the most part, for countless *n*_estimators, we utilize a more modest benefit of the learning rate. For instance, whenever our powerless classifier gets the opportunities of right expectations just somewhat more than arbitrary theory, the learning rate is 0.5. It is normal to utilize a more modest benefit of the learning rate going somewhere in the range of 0 and 1, as 0.1 and 0.001 because, in any case, it brings about the issue of overfitting.

Hyperparameters of classifier 1 (NuSVC) are represented in Table 9.

**Table 9:** Hyperparameters selection for Nu SVC.

**Hyperparameters**	**Value**	**Justification**
C	150	This value basically determines the training speed of the algorithm. Setting a value less than 100 was taking more time, whereas values above 150 showed less learning by the model. On trying many approaches, C = 150 was considered the most suitable for the model
Degree	4	Due to the 2D feature vector and many attributes of the dataset, a degree of 4 was considered better. Any value above 4 showed a similar performance on consuming more time
Kernel	RBF	According to research, the RBF kernel is more suitable for stacking multiple classifiers (13)
Gamma	auto	This is the default value

### Hyperparameter Selection for Random Forest

The hyperparameters selected for RF are illustrated in Table 10.

**Table 10:** Hyperparameter selection for RF.

**Hyperparameters**	**Value**	**Justification**
*n*_estimators	700	It determines the number of trees required to build before making average predictions. The increase in value increases the performance and decreases the speed of training. So, up to 700, the model was performing with consistently increasing accuracy and speed, but after that, the speed started to decrease
max_features	Auto	This is the default value. It improves performance at each node. The model was showing degrading performances at other values
max_depth	50	It represents the depth of each tree in the model. The more the depth, the more the information will be gained from each tree, which will add on to the info_gain parameter of the model. However, values above 50 decreased the speed of the process
min_samples_leaf	0.005	It represents the minimum sample of dataset at each level. It is always beneficial to have fewer samples at each node to prevent complexity in learning

### Hyperparameter Selection for AdaBoost

The hyperparameters selected for AdaBoost are illustrated in Table 11.

**Table 11:** Hyperparameter selection for AdaBoost.

**Hyperparameter**	**Value**	**Justification**
*n*_features	40	It represents the number of features seen during the model fit. The value was according to the number of attributes in the dataset
*n*_estimators	200	It represents the number of estimators at which the boosting terminates. Up to the value of 200, the boosting was enhanced without affecting the learning rate. However, above 200, the boosting rate became constant, leading to a decrease in the learning of the model
Learning rate	1	This is the default value. The model performed better at the initial value
Algorithm	SAMME.R	It performs faster than SAMME and achieves lower test errors with fewer boosting iterations
*N*_classes	4	The number of classes was 4

### Hyperparameter Selection for XGBOOST

The hyperparameters selected for XGBOOST are illustrated in Table 12.

**Table 12:** Hyperparameter selection for XGBOOST.

**Hyperparameter**	**Value**	**Justification**
Num_boost_round	150	It represents the number of rounds to build the model. Its optimal high value depends largely on other parameters, that is why its value is kept relatively lower to avoid changing it as and when we use other parameters
Early_stopping_rounds	20	It should generally be quite low so that we do not have to worry about improving the accuracy of the model as it has early stopping
*N*_fold	6	It is the number of folds required for cross validation. Initially, it was set to 5, and later, on observing the improvement in the increase in value, it was changed to 6. However, above 7. it did not incline toward the learning curve
Metrics	MAE	The mean absolute error allows us to compare estimates of different sequences in different scales

## Results and Discussion

### Results for the VGG16 Model

**Figure 9:**
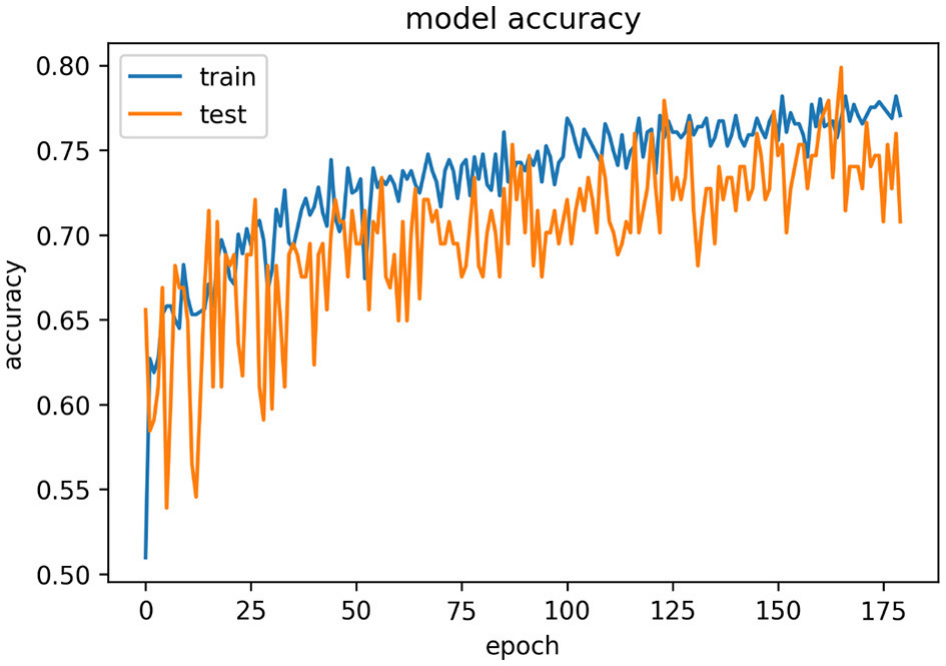
Epochs vs. accuracy graph for the VGG16 model.

Figure 9 represents the epoch vs. accuracy graph for the VGG16 model using the transfer learning approach. Further, Table 13 represents the evaluation metrics for the VGG16 model.

**Table 13:** Evaluation metrics for the VGG16 model.

**Class**	***n* (truth)**	***n* (classified)**	**Accuracy (%)**	**Precision**	**Sensitivity**	**Specificity**	**F1 score**
Class 1 (sleep)	3,205	3,653	84.5	0.81	0.88	0.856	0.84
Class 2 (hunger)	3,245	3,609	86.3	0.82	0.93	0.902	0.86
Class 3 (pain)	3,685	3,367	84.2	0.78	0.86	0.855	0.83
Class 4 (diaper)	3,551	3,057	77.8	0.74	0.83	0.81	0.77

From the above table, we can analyze that the model performs the best in classifying the cries due to hunger. The model has an imbalanced nature in prediction metrics of the other three classes. This is one of the limitations of the model as the model classifies hunger cries more even though the samples are fewer than the pain and diaper cries. Hence, the model needs to have a balance in prediction metrics among all of the classes. Tragically, there are two significant disadvantages with VGGNet: It is agonizing to prepare. The organization's engineering loads themselves are enormous (concerning circle/data transmission). Because of its profundity and number of completely associated hubs, VGG16 is over 533MB. This makes sending VGG a tedious errand. VGG16 is utilized in many profound learning picture arrangement issues; in any case, more modest organization models are frequently more alluring (like SqueezeNet, GoogLeNet, and so on). Yet, it is an extraordinary structure block for learning purposes as it is not difficult to carry out.

### Results for the YOLOv4 Model

**Figure 10:**
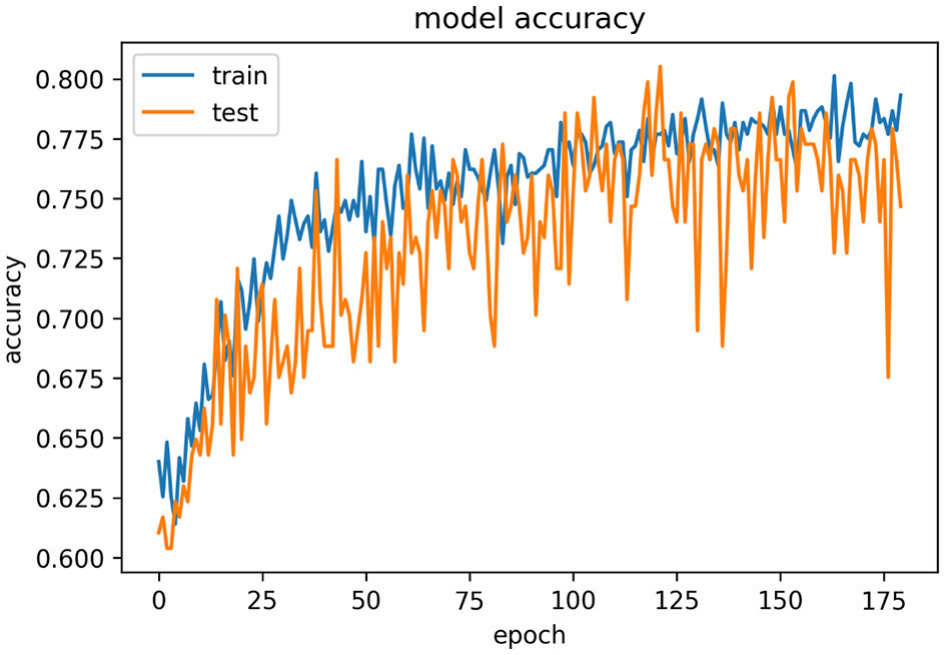
Epochs vs. accuracy graph for the YOLOv4 model.

Figure 10 represents the epochs vs. accuracy graph for the YOLOv4 model. From this graph, we can observe the improvement in results of the YOLOv4 model compared to those of the VGG16 model.

**Figure 11:**
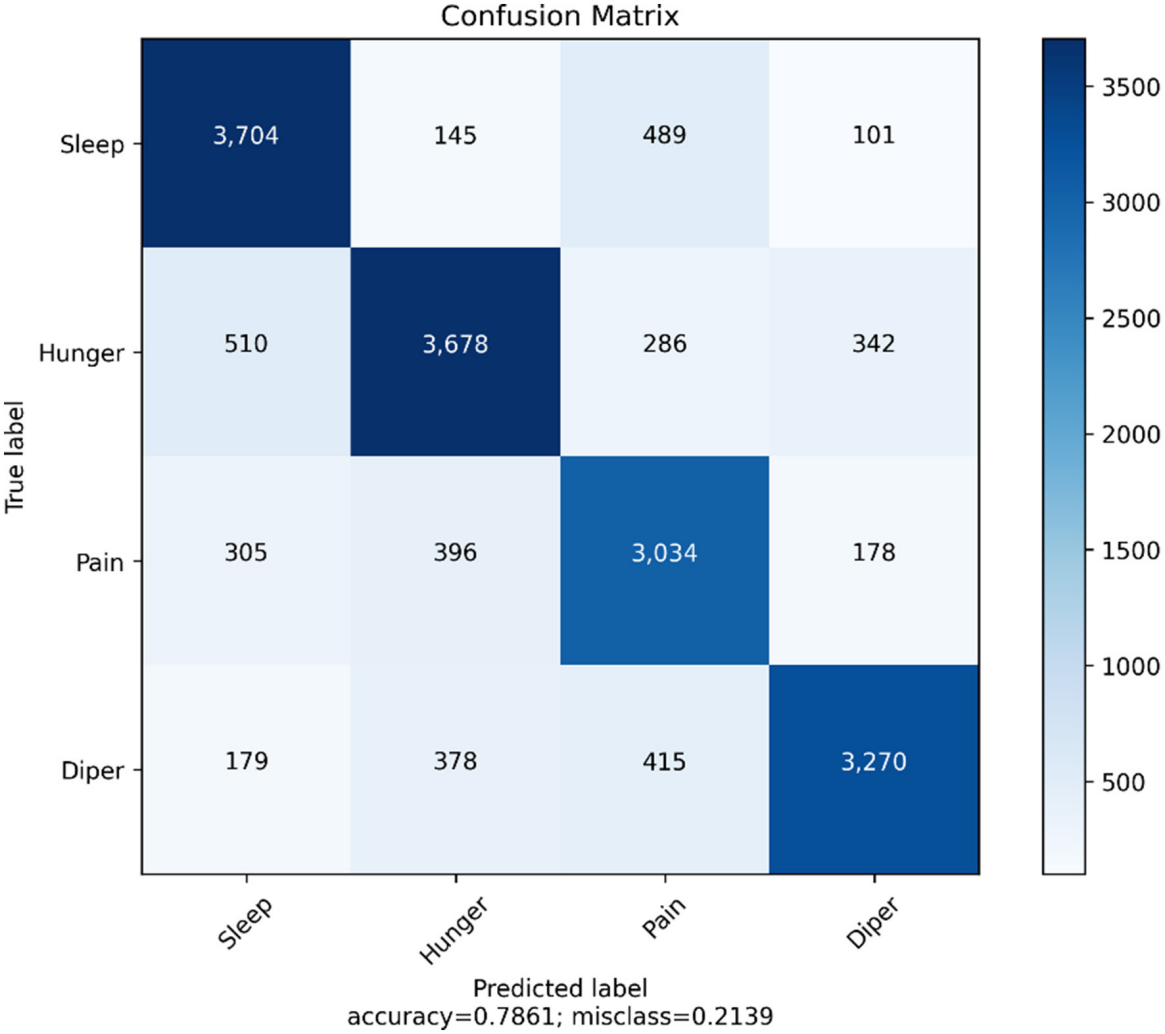
Confusion matrix for the YOLOv4 model.

We have generated the confusion matrix and the evaluation metrics for the YOLOv4 model. The main objective of developing the confusion matrix is to observe the number of true positive predictions for every class. The output consists of four different classes. They are sleep, hunger, pain, and diaper. Figure 11 illustrates the confusion matrix for the YOLO v4 model. Table 14 represents the evaluation metrics of the YOLOv4 model.

**Table 14:** Evaluation metrics for the YOLOv4 model.

**Class**	***n* (truth)**	***n* (classified)**	**Accuracy (%)**	**Precision**	**Sensitivity**	**Specificity**	**F1 score**
Class 1 (sleep)	3,205	3,678	87.8	0.83	0.90	0.83	0.85
Class 2 (hunger)	3,245	3,704	86.7	0.81	0.92	0.88	0.83
Class 3 (pain)	3,685	3,270	82.3	0.77	0.85	0.81	0.80
Class 4 (diaper)	3,551	3,034	75.6	0.74	0.84	0.79	0.77

The overall accuracy of the YOLOv4 model is 78.61%. This is better than the VGG16 model as this model is balancing its validation metrics for two classes (hunger and sleep), which accounts for 50% of the entire test data. From the cross-validation of YOLOv4, we can infer the performance of the model in predicting all four classes.

From Table 14, we can understand the evaluation metrics of the prediction classes. We can infer that sleep cries have the highest F1 score and the model works almost the same in predicting sleep and hunger cries. This is an improvement from the VGG16 model, as it was more inclined toward hunger cries. However, an imbalance still exists for the pain and diaper classes. The evaluation metrics need to be improved for these two classes. Affectability estimates how frequently a test accurately produces a positive outcome for individuals who have the condition that is being tried for (otherwise called the “genuine positive” rate). An exceptionally delicate test will hail nearly every individual who has the infection and will not create some bogus adverse outcomes. Particularity estimates a test's capacity to accurately produce an adverse outcome for individuals who do not have the condition that is being tried for (otherwise called the “genuine negative” rate). The F1 score is the weighted normal of precision and recall. Naturally, it is not as straightforward as precision; however, F1 is typically more valuable than exactness, particularly on the off chance that we have skewed class circulation.

### Results for Multistage Heterogeneous Stacking Ensemble Classification Model 2

**Figure 12:**
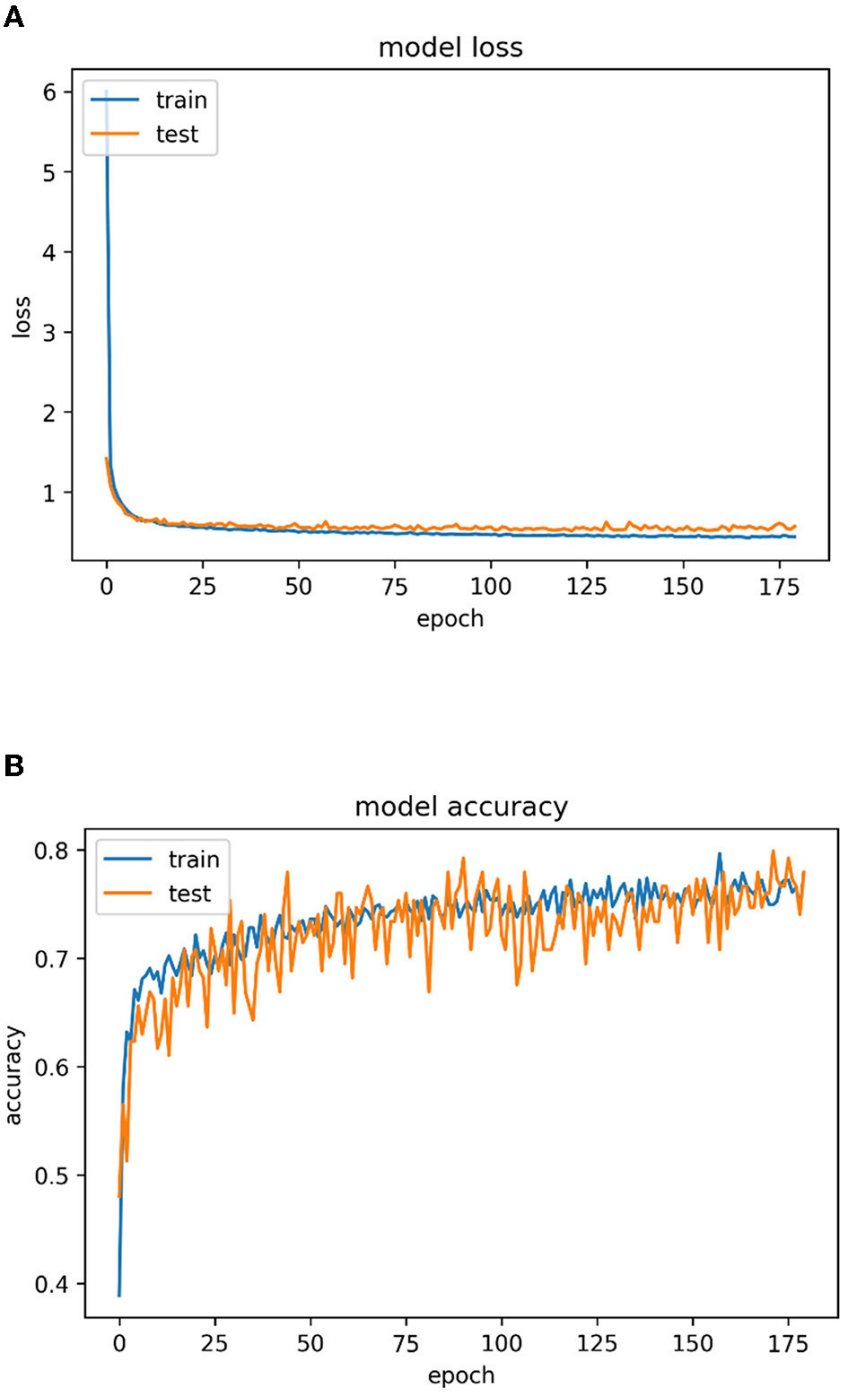
**(A)** Epoch vs. loss graph for multi-stage heterogeneous stacking ensemble classification model 2 and **(B)** epoch vs. accuracy for multistage heterogeneous stacking ensemble classification model 2.

Figure 12A represents the epoch vs. loss graph and Figure 12B illustrates the epoch vs. accuracy graph of the proposed multistage heterogeneous stacking ensemble classification model 2 for a maximum of 175 epochs.

### Exploring the Evaluation Metrics of the Proposed Multistage Heterogeneous Stacking Ensemble Classification Model 2

**Figure 13:**
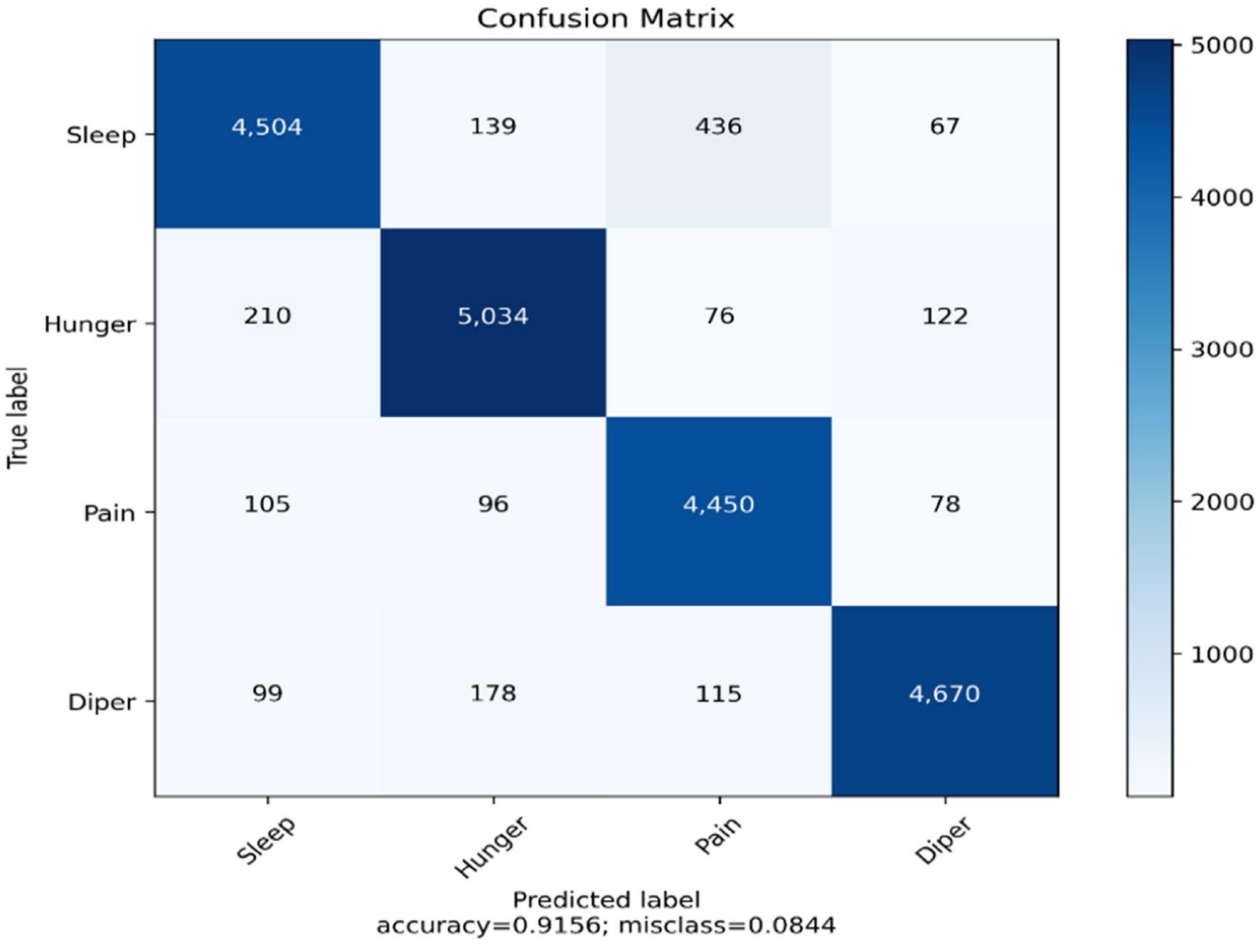
Confusion matrix of multi-tage heterogeneous stacking ensemble classification model 2.

**Figure 14:**
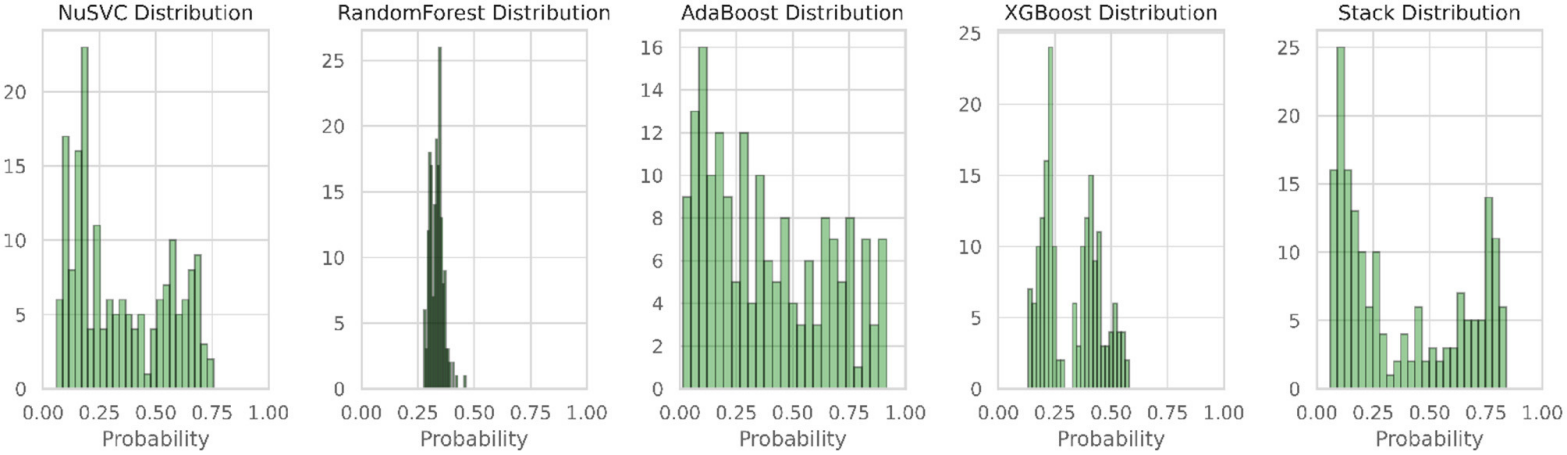
Probability distributions of the four classifiers and multistage heterogeneous stacking ensemble classification model 2.

Figure 13 illustrates the confusion matrix of the multistage heterogeneous stacking ensemble classification model 2, and Figure 14 portrays the probability distributions of the four classifiers along with the probability distribution of multi-stage heterogeneous stacking ensemble classification model 2.

From the cross-validation of the proposed model, we can infer that the model is performing at its best in predicting all of the classes. A perfect balance is maintained between all of the prediction labels. The overall accuracy of the model is 91.56%, which is far better than the hypertuned and optimized YOLOv4 model. The accuracies of all of the classes have also been increased compared to the previously implemented CNN variants.

Table 15 represents the evaluation metrics of the proposed multistage heterogeneous stacking ensemble classification model 2. By observing the computed values of the different metrics, we can infer that it has performed much better than the top CNN variants. The overall validation accuracy produced by the model is around 92%. With respect to the computational time, it was almost four times faster than CNN variants due to the advantage of highly advanced ensemble algorithms.

**Table 15:** Multistage heterogeneous stacking ensemble classification model 2.

**Class**	**Accuracy (%)**	**Precision**	**Sensitivity**	**Specificity**	**F1 score**
Class 1 (sleep)	94.5	0.87	0.88	0.87	0.93
Class 2 (hunger)	96.3	0.95	0.93	0.89	0.95
Class 3 (pain)	91.2	0.92	0.86	0.80	0.91
Class 4 (diaper)	92.8	0.94	0.83	0.76	0.90

From Table 15, we can infer that all of the classes are classified with the best precision values after performing many hypertuning techniques. The hunger cries are performing the best compared to all other classes. The F1 scores of all of the classes have been improved compared to the previous two models. The proposed algorithm provides a balance in classifying the results of all of the prediction classes.

### Plotting and Analyses of the Receiver Operator Characteristic Curve for the Four Prediction Classes

The area under ROC curve (AUC) is a valuable metric for assessing the nature of class intervals for delicate classifiers. In the multiclass setting, we can envision the exhibition of multiclass models as indicated by their one-vs.-all accuracy review curves. The AUC can likewise be summed up to the multiclass setting.

**Figure 15:**
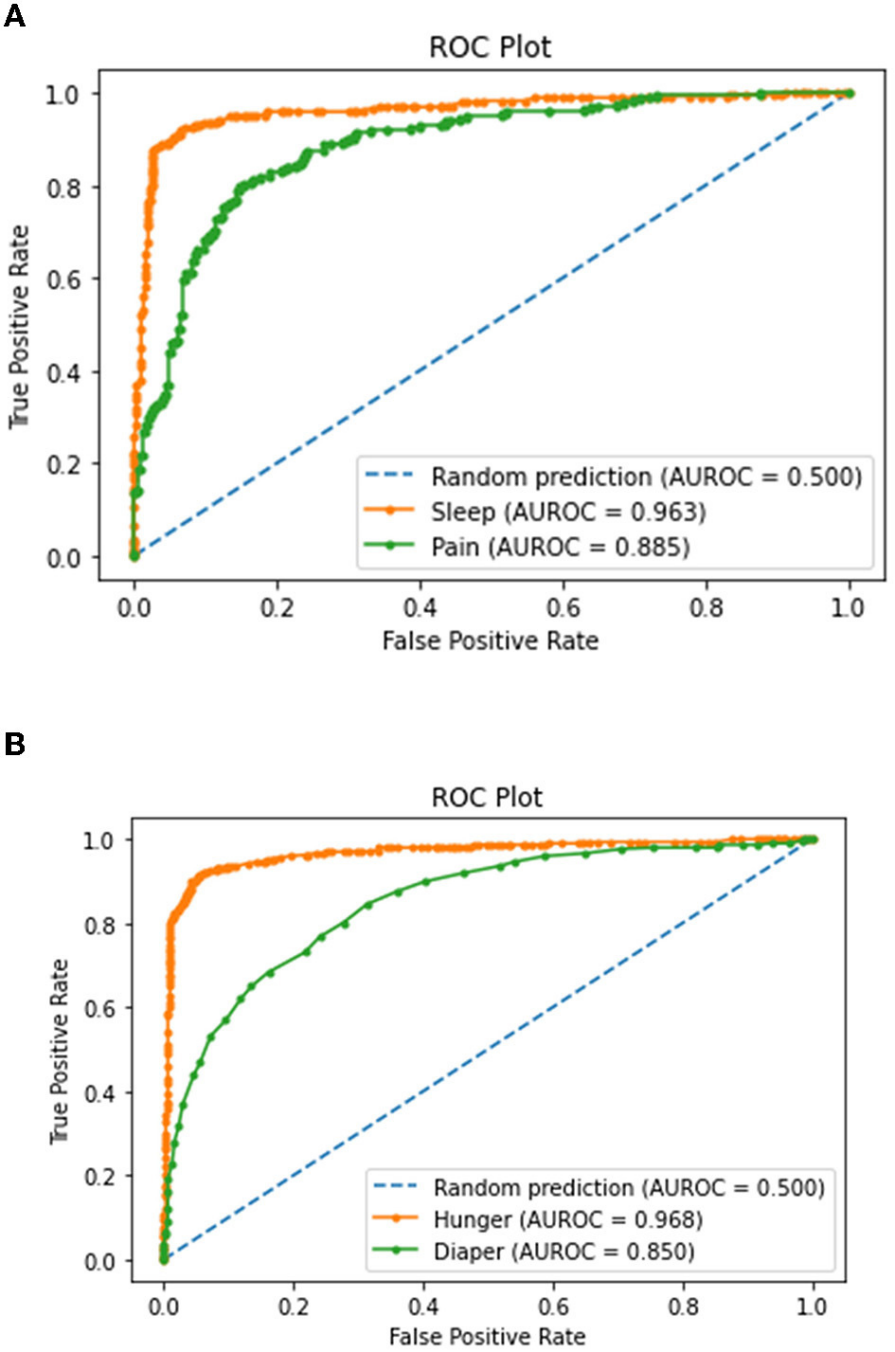
Receiver operator characteristic (ROC) curve for **(A)** sleep and pain classes and **(B)** hunger and diaper classes.

Figure 15A represents the receiver operator characteristic (ROC) curve and AUC scores for the sleep and pain prediction classes. From this, we can infer that the sleep class is getting predicted more accurately and perfectly compared to the pain class. This is because the higher the AUC score, the better the performance of the model in differentiating the true and false outcomes for the particular class. The AUC score for the sleep class is 0.963, and for the pain class is 0.885. From Figure 15B, we can infer that the ROC curve and AUC scores for the hunger class are much better than those for the diaper class. This is also because the model is more inclined toward the hunger class as compared to the diaper class.

## Discussion

The proposed hybrid model performed much better compared to the previously mentioned and implemented CNN variants in terms of accuracy and computational time. It achieved an overall accuracy of 92%. The stacking approach is highly suitable for the multiclass classification problem. Since the dataset consisted of audio signals and four different output prediction classes, consideration of four different classifiers simultaneously was highly beneficial. All of the classifiers were dependent on one another, unlike the top CNN variants. Due to this, the overall architecture of the model is more complex than the CNN variants. The mean AUC score of the proposed hybrid model is approximately 92%.

### Comparison With State-of-the-Art Models

Table 16 presents the comparison of the proposed multistage heterogeneous stacking ensemble classification model 2 with the state-of-the-art models. The superior performance of the proposed model 2 in terms of the mean F1 score, could be witnessed from this table.

**Table 16:** Comparison with the state-of-the-art models.

**Reference**	**Year**	**Method**	**Dataset**	**Number of emotion classes**	**Emotion classes**	**Mean F1 score**
—	—	Multistage heterogeneous stacking ensemble model (proposed model)	Infant cry dataset—National Taiwan University Hospital Yunlin Branch, Taiwan	4	Diaper, sleep, hunger, pain	0.923
Jian et al. (33)	2021	LSTM + deepf_3	Fau Aibo children's emotion corpus database	5	Angry, hungry, pain, sad, tired	0.604
Ashwini et al. (4)	2021	Linear support vector machine	Infant cry dataset—National Taiwan University Hospital Yunlin Branch, Taiwan	3	Hunger, pain, sleepy	0.844
Jiang et al. (34)	2021	Gaussian mixture model-universal background model	Donate-A-Cry corpus	4	Hungry, discomfort, scared, tired	0.828
Boersma et al. (35)	2021	Convolutional neural net with self-attention	CRIED dataset	3	Fussing, screaming, high-pitched screeching	0.797

## Conclusions

The multistage heterogeneous stacking ensemble model two consisting of four highly advanced boosting algorithms was roughly able to predict the classes on the basis of the feature vectors present in the spectrogram of the audio signals. However, these boosting algorithms are sensitive to some outliers. Our dataset comprises only four major reasons for the baby's cry. In the future, if some other major reason arises, then the model may not identify it and would produce incorrect results as it is a supervised learning model. The classifiers are trained on the feature vectors and these four classes. They will not be able to identify a new class, and this would require the model to undergo training once again to meet the updated changes. The proposed model can be extended to a semi-supervised or unsupervised approach by using some pretrained weights in a similar stacking-based model. This would save some time and efficiently provide better outcomes in terms of computational time and accuracy. In this research article, we were able to predict the reason for the infant's cry using two different approaches. In the first approach, we used pretrained weights and transfer learning approaches to predict the results. These included various CNN variants. A complete analysis and comparison were performed on the top CNN models. However, in the second approach, we made use of the multistage heterogeneous stacking ensemble classification model for enhancing the performance of the model using boosting algorithms. This approach produced much better results in terms of computational time, power, and accuracy.

## Data Availability Statement

The original contributions presented in the study are included in the article/supplementary material, further inquiries can be directed to the corresponding author.

## Ethics Statement

The studies involving human participants were reviewed and approved by Human Research Ethics Committee at National Cheng Kung University (NCKU HREC) authorized by Ministry of Education, Taiwan. Written informed consent to participate in this study was provided by the participants' legal guardian/next of kin.

## Author Contributions

C-YC conceptualized and supervised the research. C-YC carried out funding acquisition. VJ investigated the data, performed the methodology, and implemented the software code. KS and C-YC carried out the project administration and validated the results. VJ and KS wrote the manuscript. VJ, KS, PV, and VR reviewed and edited the manuscript. All authors contributed to the article and approved the submitted version.

## Funding

This work was funded by the Ministry of Science and Technology, Taiwan (MOST 109-2221-E-224-045-MY3) and the Ministry of Education: Higher Education Sprout Project.

## Conflict of Interest

The authors declare that the research was conducted in the absence of any commercial or financial relationships that could be construed as a potential conflict of interest.

## Publisher's Note

All claims expressed in this article are solely those of the authors and do not necessarily represent those of their affiliated organizations, or those of the publisher, the editors and the reviewers. Any product that may be evaluated in this article, or claim that may be made by its manufacturer, is not guaranteed or endorsed by the publisher.
